# Screening and Analysis of the Marker Components in *Ganoderma lucidum* by HPLC and HPLC-MS^n^ with the Aid of Chemometrics

**DOI:** 10.3390/molecules22040584

**Published:** 2017-04-06

**Authors:** Lingfang Wu, Wenyi Liang, Wenjing Chen, Shi Li, Yaping Cui, Qi Qi, Lanzhen Zhang

**Affiliations:** School of Chinese Materia Medica, Beijing University of Chinese Medicine, Beijing 100102, China; fanglingwu@163.com (L.W.); lwy1054289310@163.com (W.L.); sdcwjing@163.com (W.C.); lishi816@126.com (S.L.); 20150931830@bucm.edu.cn (Y.C.); cici_jiayou@163.com (Q.Q.)

**Keywords:** *Ganoderma lucidum*, triterpenes, HPLC-MS^n^, Similarity Analysis (SA), chemometrics

## Abstract

*Ganoderma* triterpenes (GTs) are the major secondary metabolites of *Ganoderma lucidum*, which is a popularly used traditional Chinese medicine for complementary cancer therapy. The present study was to establish a fingerprint evaluation system based on Similarity Analysis (SA), Cluster Analysis (CA) and Principal Component Analysis (PCA) for the identification and quality control of *G. lucidum*. Fifteen samples from the Chinese provinces of Hainan, Neimeng, Shangdong, Jilin, Anhui, Henan, Yunnan, Guangxi and Fujian were analyzed by HPLC-PAD and HPLC-MS^n^. Forty-seven compounds were detected by HPLC, of which forty-two compounds were tentatively identified by comparing their retention times and mass spectrometry data with that of reference compounds and reviewing the literature. Ganoderic acid B, 3,7,15-trihydroxy-11,23-dioxolanost-8,16-dien-26-oic acid, lucidenic acid A, ganoderic acid G, and 3,7-oxo-12-acetylganoderic acid DM were deemed to be the marker compounds to distinguish the samples with different quality according to both CA and PCA. This study provides helpful chemical information for further research on the anti-tumor activity and mechanism of action of *G. lucidum*. The results proved that fingerprints combined with chemometrics are a simple, rapid and effective method for the quality control of *G. lucidum*.

## 1. Introduction

*Ganoderma lucidum* (Leyss. ex Fr.) Karstis is one of the most highly used medicinal fungi in the world. Its fruiting body, called lingzhi or reishi, has been widely used in traditional Chinese medicine (TCM) as a dietary supplement and medicinal herb in China and other eastern countries. Modern medical research has indicated that *G. lucidum* has comprehensive biological activities, such as anti-cancer [1,2,3,4,5], immune-modulating [1,3,6], anti-oxidant [6,7,8], anti-microbial [9], anti-inflammatory [10], anti-HIV-1 [11], and so on, among which the most attractive is its anti-cancer activity.

To date, more than 400 compounds were isolated and identified from *G. lucidum*. Over 150 compounds such as ganoderic acid A (GA-A), GA-C_2_, GA-D, GA-DM, GA-lactone, ganoderiol F, ganodermanotriol and so on belong to the *Ganoderma* terpene (GT) class which are regarded as the main medicinal components [9,12,13,14,15]. Accumulating evidence has shown that GTs can inhibit the proliferation of hepatoma cells and HeLa cells, as well as human colon cancer cells HT-29 [16,17,18]. The type and content of triterpene acids reflects the quality of *G. lucidum*, so GTs could be used as marker components to evaluate the quality of *G. lucidum*.

The therapeutic effects of traditional Chinese medicines (TCMs) are based on the complex interactions of numerous complicated chemical constituents as a whole system, so methods are needed in order to control the quality of this complex system. In this case, HPLC fingerprints of key components provide a new approach for quality control of traditional Chinese medicines. There are many studies about fingerprints analysis combined with chemometrics for the quality control of traditional Chinese medicines and to find the bioactive components [19,20,21].

Some studies on the fingerprints of *G. lucidum* have been reported [22,23,24,25], but in these studies, only a few compounds were identified by HPLC-MS^n^. Yang [26] focused on chemical identification of the GTs, and identified thirty-two compounds, but no marker compounds were found from cluster analysis (CA) and principal component analysis (PCA).

In the present study, forty-seven peaks were detected in HPLC-PDA, of which thirty-seven were common peaks in the similarity analysis. Forty-two known triterpenoids were identified by high-resolution liquid mass spectrometry. To the best of our knowledge, this is the first time that so many compounds were identified. We also found for the first time that ganoderic acid B, 3,7,15-trihydroxy-11,23-dioxo-lanost-8,16-dien-26-oic acid, lucidenic acid A, ganoderic acid G, and 3,7-oxo-12-acetylganoderic acid DM might be suitable marker compounds to distinguish between *G. lucidum* samples of different quality, according to CA and PCA. This study provides helpful chemical information for further research on the anti-tumor activity and mechanism of action of *G. lucidum*. The method developed in our study also provides a scientific foundation for the quality control of *G. lucidum*.

## 2. Results and Discussion

### 2.1. Validation of the Method

The relative retention time, relative peak area and similarities were used to evaluate the quality of the fingerprints. Dehydrotumulosic acid (peak 15) which is a large single peak in the middle of the chromatogram, was assigned as the reference peak to calculate relative retention times and relative peak areas.

The precision was determined by repeated injection of the same sample solution six consecutive times. The RSDs of relative retention time and relative peak area of the common peaks were all below 0.94% and 2.88%, respectively; the similarities of different chromatograms were all above 0.995.

The repeatability was evaluated by the analysis of six prepared samples. The RSDs of relative retention time and relative retention time of the common peaks were all below 0.95% and 2.86%, respectively; the similarities of different chromatograms were all above 0.995.

Stability testing was performed with one sample over 24 h. The RSDs of relative retention time and relative retention time of the common peaks were all below 1.06% and 2.71%; the similarities of different chromatograms were all 1.000. All these results indicated that the samples remained stable during the testing period and the conditions were satisfactory for the fingerprint analysis.

### 2.2. Similarity Analysis (SA)

The chromatographic profile must be representative of all the samples and have the features of integrity and fuzziness. By analyzing the mutual pattern of chromatograms, the identification and authentication of the samples can be conducted well even if the amounts of some chemical constituents are different from the others.

**Figure 1:**
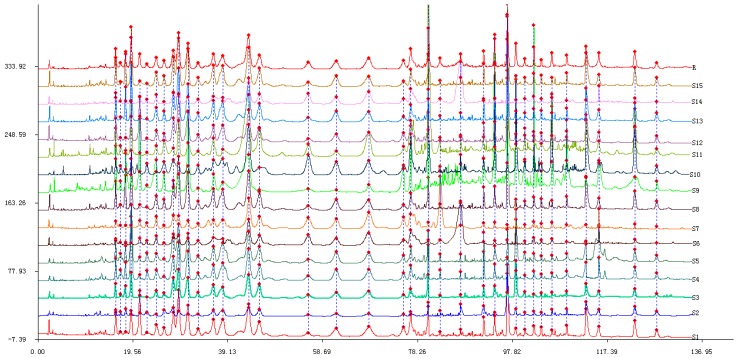
Overlaid HPLC chromatograms of samples from No. S1 to S15. The common pattern (marked R) was obtained by using the Similarity Evaluation System (SES) for the Chromatographic Fingerprints of TCMs.

Fifteen batches of samples from different habitats were determined and the chromatograms were analyzed by SES to generate a common pattern R (Figure 1). The peak area of the common peaks was list in the supplementary materials. SES for Chromatographic Fingerprint was performed to calculate the similarities of different chromatograms compared to the common pattern. The results are shown in Table 1.

The conclusion can be drawn from the results that the similarities of different chromatograms compared to the common pattern are all above 0.800, except for samples S7 (0.791) and S9 (0.772), which indicates that the chemical constituents of different samples are not highly influenced by their sources. The common pattern is a very positive identification for the samples of *G. lucidum*.

### 2.3. Identification of the Compounds Present

**Figure 2:**
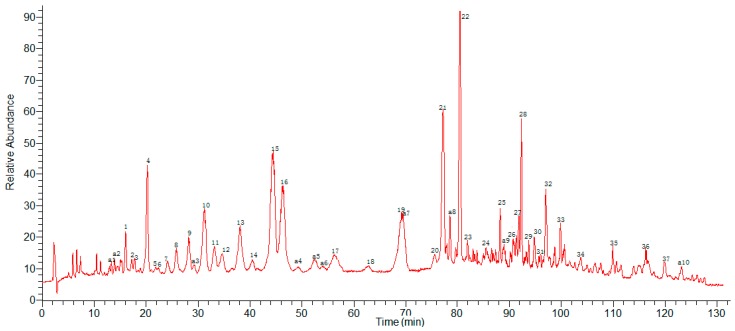
HPLC chromatograms of *G. lucidum*.

**Figure 3:**
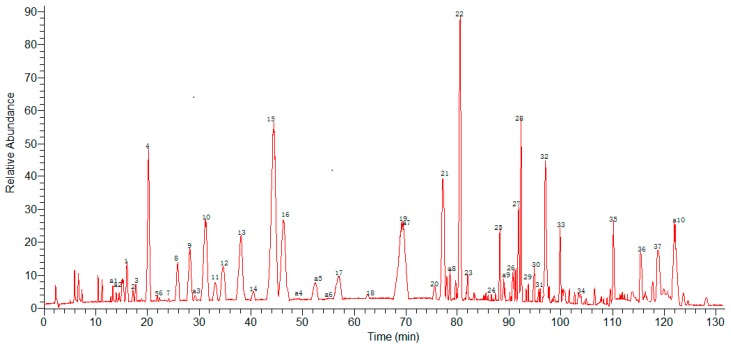
Negative mode of the HPLC-MS^n^ chromatograms of *G. lucidum*.

**Figure 4:**
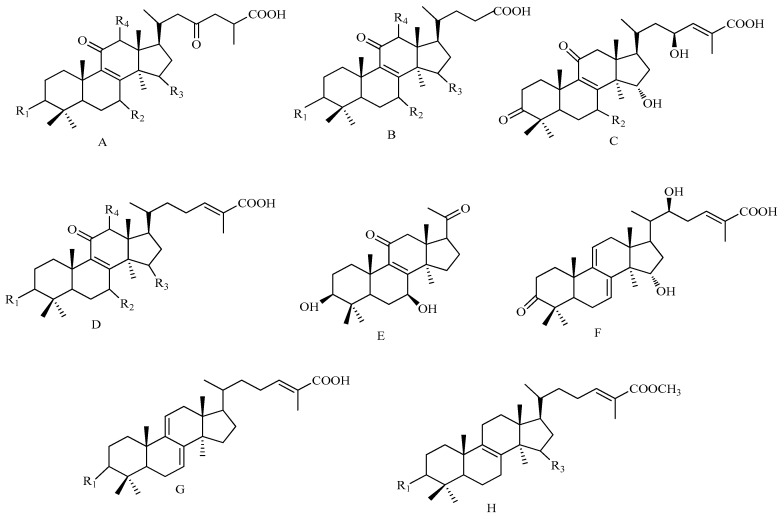
The chemical structures of the identified compounds.

HPLC-ESI-MS^n^ method was employed to identify the components in *G. lucidum* (Figure 2 and Figure 3) Molecular weights and fragmentation information (Table 2 and Table 3) were obtained. The possible structures of 37 common peaks and ten other peaks a1–a10 were deduced, as shown in Figure 4. Under the optimized MS conditions, the negative mode was used to identify the peaks.

As shown in Table 2, in the negative mode ESI-MS spectra, the [M − H]^−^ and [M − H_2_O − H]^−^ ions were found for all 47 compounds. The [M − CO_2_ − H]^−^ ion was seen for most of the compounds. In type A and C, the molecular weight of pyrolysis fragments of D ring was 194, while there is a^Δ^20, 22 or ^Δ^16, 17, the molecular weight of pyrolysis fragments of D ring was 192. In type B, the molecular weight of pyrolysis fragments of D ring was 138. In type D, the molecular weight of pyrolysis fragments of D ring was 178. In type E, the molecular weight of pyrolysis fragments of D ring was 80, only for compound **34**. In type F, the molecular weight of pyrolysis fragments of D ring was also 194, without R_1_, R_2_, R_3_, and R_4_, only for compound **35**. In type G, the molecular weight of pyrolysis fragments of D ring was also 178, without R_2_, R_3_, and R_4_, only for compound **35**. In type H, the molecular weight of pyrolysis fragments of D ring was also 192, without C=C, only for compound **a2**.

### 2.4. Cluster Analysis (CA)

Cluster analysis is a multivariate analysis technique that is used to sort samples into groups. It is widely applied for fingerprint analysis, because it is a nonparametric data interpretation method and simple to use. CA provides a visual representation of complex data. Average linkage between groups was applied, and Pearson correlation was selected as a measurement. The method can classify different herbs by measuring the peak areas from their corresponding HPLC fingerprints. The common characteristic peaks, which were calculated by the Similarity Evaluation System, were selected for the CA. Cluster analysis of *G. lucidum* samples was performed based on the relative peak areas of all 37 common peaks.

**Figure 5:**
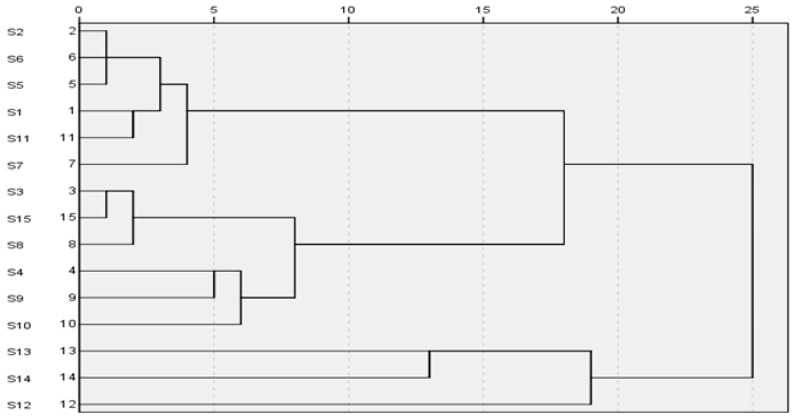
Results of cluster analysis of 15 samples.

The CA results are shown in Figure 5, where the quality characteristics are revealed more clearly. The cluster analysis results show that the samples could be divided into three quality clusters. Among them, Cluster I includes the samples S2, S6, S5, S1, S11 and S7, Cluster III includes S13 S14 and S12, the others are in Cluster II. All the compounds in Cluster II had much lower concentrations than the other two clusters.

Cluster I was distinguished as it contains more 3-acetylganoderenic acid K (**F3**), ganoderic acid G (**F9**), ganoderic acid B (**F10**), unknown **F11**, lucidenic acid A (**F14**), and 3,7-oxo-12-acetylganoderic acid DM (**F30**) than Clusters II and III. The higher concentration of these compounds in Cluster I may be due to the good quality of *G. lucidum* herb. This indicated that these compounds could be used as marker compounds to distinguish the *G. lucidum* samples with different quality. The results of CA could be validated against each other and provided more references for the quality evaluation of *G. lucidum*.

### 2.5. Principal Components Analysis (PCA)

**Figure 6:**
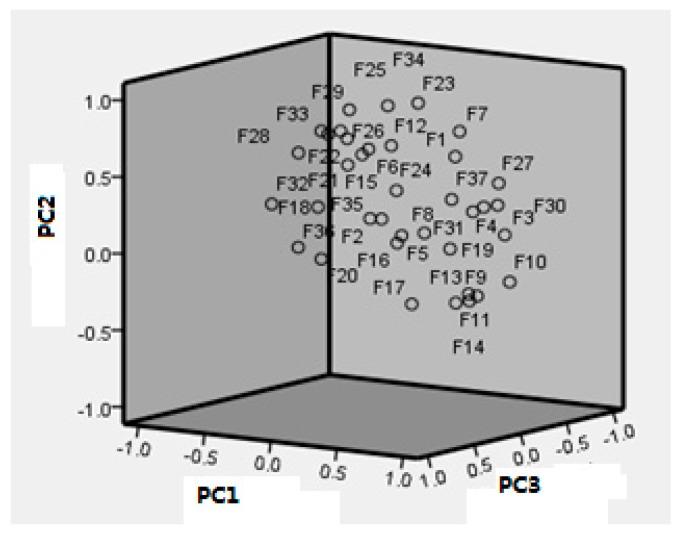
PCA scores plots of the sample from different regions.

To evaluate the variations in quality of the 15 samples, PCA was carried out with the relative amounts of each identified component. The contents of 37 fingerprint peaks were applied to evaluate the sample variations. Figure 6 shows the score plots obtained by PCA. The first six principal components accounted for 93.69% of the total variance. Examination of the score plots indicates that the main components responsible for the separation were ganoderic acid B (**F10**), 3-acetylganoderenic acid K (**F3**), 3,7-oxo-12-acetylganoderic acid DM (**F30**), ganoderic acid G (**F9**), 3,7,15-trihydroxy-11,23-dioxolanost-8,16-dien-26-oic acid (**F4**), lucidenic acid A (**F14**), 3-acetyl-ganoderic acid H (**F27**) and unknown **F11**, as shown in Figure 6 and Table 4.

These components were deemed to be the marker compounds of sample variation. This result is in accord with the one obtained from the cluster analysis (CA). The combination of PCA and CA was thus a useful tool for quality control and evaluation of *G. lucidum*.

## 3. Materials and Methods

### 3.1. Samples and Reagents

Fifteen *G. lucidum* samples were purchased from different regions of China and authenticated by Professor Chun-Sheng Liu (School of Chinese Materia Medica, Beijing University of Chinese Medicine, Beijing, China). Each sample (three replicates) was placed in a dark and dry environment. The regions where the 15 samples were obtained are listed in Table 5. HPLC grade acetonitrile and acetic acid were obtained from Fisher (Waltham, MA, USA); distilled water was bought from Watsons (Beijing, China) and was filtered through a 0.22 µm membrane (Dikma, Beijing, China) prior to use. All other reagents were of analytical grade.

### 3.2. Sample Preparation

Dried powder of *G. lucidum* from different regions (1 g) was accurately weighed out and transferred into a 100 mL conical flask. Chloroform (50 mL) was added to the flask and the flask with the chloroform and powder was placed in an ultrasonic extraction device and extracted for 30 min twice. The solution was cooled and filtered through filter paper, and then the solvent was recovered using a rotary evaporator. The residue was dissolved in a 10 mL volumetric flask using methanol. The solution was filtered through a 0.22 µm membrane filter for fingerprint analysis.

### 3.3. Apparatus and Parameters

A Waters Alliance HPLC 2695 series instrument (Waters, Manchester, UK) was used to perform the high performance liquid chromatography (HPLC) analysis. Mobile phase: A (acetonitrile); B (H_2_O:CH_3_COOH, 100:0.2, *v*/*v*). Column: Agilent C18 (250 mm × 4.6 mm, 5 μm), maintained at 30 °C with flow rate of 1.0 mL·min^−1^. The detection wavelength was set at 254 nm for acquiring chromatograms. The injection volume was 20 µL. Gradient elution procedure: 0 min (20 % A) → 8 min (29% A) → 25 min (29% A) → 55 min (30% A) → 65 min (30% A) → 70 min (31% A) → 90 min (65% A) → 110 min (90% A) → 135 min (90% A).

The LCMS-IT-TOF instrument (Shimadzu, Kyoto, Japan) was equipped with an ESI source used in negative ionization mode. The interface and MS parameters were as follows: nebulizer pressure, 100 kPa; dry gas, N_2_ (1.5 L/min); drying gas temperature, 200 °C; spray capillary voltage, 4000 V; scan range, *m*/*z* 100–1000. Mobile phase: A (acetonitrile); B (H_2_O:CH_3_COOH, 100:0.2, *v*/*v*). Column: Agilent C18 (250 mm × 4.6 mm, 5 μm), maintained at 30 °C with flow rate of 1.0 mL·min^−1^. The injection volume was 20 µL. Gradient elution procedure: 0 min (20 % A) → 8 min (29% A) → 25 min (29% A) → 55 min (30% A) → 65 min (30% A) → 70 min (31% A) → 90 min (65% A) → 110 min (90% A) → 135 min (90% A).

### 3.4. Statistical Analyses

The HPLC data were used for fingerprint analysis and chemometrics. HPLC-MS^n^ was used for identification of the 47 peaks. Cluster analysis (CA) and principal components analysis (PCA) were performed by SPSS (SPSS statistical software package, version 20.0, SPSS Inc., Chicago, IL, USA).

## 4. Conclusions

The therapeutic effects of traditional Chinese medicines (TCM) are based on the complex interactions of complicated chemical constituents as a whole system. HPLC and HPLC-MS^n^ fingerprint analysis combined with chemometrics were employed to study the complex *G. lucidum* system. According to previous extensive phytochemical and pharmacological studies, triterpenoid acids were the most important chemical components in the samples, which had a variety of potential biological activities. The qualitative analysis and quantification of triterpenoid acids can better reflect the therapeutic effects and quality of *G. lucidum*. The chromatographic method is predominant to control the quality and stability of the complex system. This study provided a systematic method for the quality control of *G. lucidum* by HPLC fingerprinting and the HPLC-MS^n^ evaluation system based on Similarity Analysis (SA), Cluster Analysis (CA) and Principal Component Analysis (PCA). As a result, a common mutual pattern was established by determining and comparing the fingerprints of 15 samples of *G. lucidum* from different regions. Forty-seven compounds were detected by HPLC-MS^n^, of which forty-two compounds were tentatively identified by comparing their retention times, and mass spectrometry data with that of reference compounds and literature data. Ganoderic acid B (**10**), 3,7,15-trihydroxy-11,23-dioxo-lanost-8,16-dien-26-oic acid (**F4**), Lucidenic acid A (**F14**), Ganoderic acid G (**F9**), unknown (**F11**), 3,7-oxo-12-acetylganoderic acid DM (**F30**) were deemed to be the markers to distinguish *G. lucidum* samples of different quality. The proposed method can be used to improve the quality control of *G. lucidum*, thus ensuring the effectiveness of *G. lucidum* herbs. There are still five peaks—**2**, **11**, **28**, **31** and **a10**—which were not identified by HPLC-MS^n^, of which compound **11** were used as marker compound to distinguish the *G. lucidum* of different quality. These components require further study.

## Figures and Tables

**Table 1:** The results of similarities of the chromatograms from different origins.

No.	S1	S2	S3	S4	S5	S6	S7	S8	S9	S10	S11	S12	S13	S14	S15	R
S1	1.000	0.820	0.925	0.848	0.799	0.723	0.701	0.921	0.699	0.692	0.748	0.714	0.774	0.708	0.723	0.935
S2	0.820	1.000	0.831	0.733	0.707	0.673	0.636	0.803	0.670	0.803	0.797	0.777	0.624	0.642	0.687	0.864
S3	0.925	0.831	1.000	0.914	0.853	0.795	0.768	0.961	0.663	0.735	0.813	0.833	0.728	0.785	0.838	0.965
S4	0.848	0.733	0.914	1.000	0.877	0.711	0.676	0.911	0.597	0.672	0.744	0.674	0.604	0.694	0.509	0.907
S5	0.799	0.707	0.853	0.877	1.000	0.659	0.622	0.853	0.562	0.618	0.680	0.651	0.671	0.636	0.689	0.857
S6	0.723	0.673	0.795	0.711	0.659	1.000	0.843	0.728	0.509	0.739	0.744	0.653	0.481	0.984	0.648	0.825
S7	0.701	0.636	0.768	0.676	0.622	0.843	1.000	0.706	0.512	0.669	0.695	0.665	0.705	0.862	0.642	0.791
S8	0.921	0.803	0.961	0.911	0.853	0.728	0.706	1.000	0.664	0.697	0.784	0.913	0.695	0.719	0.733	0.956
S9	0.699	0.670	0.663	0.597	0.562	0.509	0.512	0.664	1.000	0.675	0.665	0.714	0.774	0.500	0.723	0.772
S10	0.692	0.803	0.735	0.672	0.618	0.739	0.669	0.697	0.675	1.000	0.799	0.650	0.711	0.720	0.686	0.826
S11	0.748	0.797	0.813	0.744	0.680	0.744	0.695	0.784	0.665	0.799	1.000	0.651	0.671	0.708	0.689	0.874
S12	0.714	0.777	0.833	0.674	0.651	0.653	0.665	0.913	0.714	0.650	0.651	1.000	0.695	0.505	0.733	0.867
S13	0.774	0.624	0.728	0.604	0.671	0.481	0.705	0.695	0.774	0.711	0.671	0.695	1.000	0.681	0.742	0.854
S14	0.708	0.642	0.785	0.694	0.636	0.984	0.862	0.719	0.500	0.720	0.708	0.505	0.681	1.000	0.554	0.810
S15	0.723	0.687	0.838	0.509	0.689	0.648	0.642	0.733	0.723	0.686	0.689	0.733	0.742	0.554	1.000	0.863
R	0.935	0.864	0.965	0.907	0.857	0.825	0.791	0.956	0.772	0.826	0.874	0.867	0.854	0.810	0.863	1.000

**Table 2:** The HPLC-MS^n^ data and compound names of the 47 peaks.

Peak No.	*t*_R_ (min)	[M− H]^−^	Negative Mode	Identification
**1**	16.07	533.3109	MS^1^：533.3109 [M − H]^−^ MS^2^：533.3109→515.3029 [M − H − 18(H_2_O)]^−^, 485.2977 [M − H − 18(H_2_O) − 30(2CH_3_)]^−^ MS^3^：515.3029→497.3448 [M − H − 18(H_2_O) − 18(H_2_O)]^−^, 303.1085 [M − H − 18(H_2_O) − 18(H_2_O) − 194(pyrolysis fragments of D ring)]^−^ 485.2977→467.3855 [M − H − 18(H_2_O) − 30(2CH_3_) − 18(H_2_O)]^−^	12-hydroxyganoderic C_2_ [26,27]
**2**	17.39	515.3452	MS^1^：515.3452 [M − H]^−^	Unknown
**3**	17.79	613.2977	MS^1^：613.2977 [M − H]^−^ MS^2^：613.2977→595.3029 [M − H − 18(H_2_O)]^−^, 553.3198[M − H − 18(H_2_O) − 42(CH_2_=CO)]^−^ MS^3^：553.3198→535.2648 [M − H − 18(H_2_O) − 42(CH_2_=CO) − 18(H_2_O)]^−^ 343.1749 [M − H − 18(H_2_O) − 192(pyrolysis fragments of D ring)]^−^	3-acetylganoderenic acid K [26]
**4**	20.22	515.3011	MS^1^：515.3011 [M − H]^−^ MS^2^：515.3011→497.9281 [M − H − 18(H_2_O)]^−^, 453.2738[M − H − 18(H_2_O) − 44(CO_2_)]^−^ MS^3^：453.2738→438.2719 [M − H − 18(H_2_O) − 44(CO_2_) − 15(CH_3_)]^−^, 423.2209[M − H − 18 (H_2_O) − 44(CO_2_) − 30(2CH_3_)]^−^, 497.9281→305.2222 [M − H − 18(H_2_O) − 192(pyrolysis fragments of D ring)]^−^	3,7,15-trihydroxy-11,23-dioxo-lanost-8,16-dien-26-oic acid [28]
**5**	21.84	517.3159	MS^1^：517.3159 [M − H]^−^ MS^2^：517.3159→499.3881 [M − H − 18(H_2_O)]^−^, 481.3099[M − H − 36(2H_2_O)]^−,^ 455.4148[M − H − 18(H_2_O) − 44(CO_2_)]^−^, 437.4261[M − H − 36(2H_2_O) − 44(CO_2_)]^−^ MS^3^：499.3881→481.3099 [M − H − 18(H_2_O) − 18(H_2_O)]^−^, 481.3099→287.2234 [M − H − 18(H_2_O) − 194(pyrolysis fragments of D ring)]^−^	Ganoderic acid C_2_ [26,29,30]
**6**	22.83	501.3214	MS^1^：501.3214 [M − H]^−^ MS^2^：501.3214→483.3465 [M − H − 18(H_2_O)]^−^, 439.4045 [M − H − 18(H_2_O) − 44(CO_2_)]^−^, 421.3404[M − H − 36(2H_2_O) − 44(CO_2_)]^−^, 289.1908 [M − H − 18(H_2_O) − 194(pyrolysis fragments of D ring)]^−^	Ganolucidic acid B [26]
**7**	24.10	457.2592	MS^1^：457.2592 [M − H]^−^ MS^2^：457.2592→ 438.9782 [M − H − 18(H_2_O) − H]^−^, 420.9395[M − H − 36(2H_2_O) − H]^−^, 413.1963[M − H − 44(CO_2_)]^−^, 397.1818 [M − H − 44(CO_2_) − 16(CH_4_)]^−^, 395.1743[M − H − 44(CO_2_) − 18H_2_O]^−^, 303.0224 [M − H − 138(pyrolysis fragments of D ring) − 16(CH_4_)]^−^	3-hydroxy-4,4,14-trimethyl-7,11,15-trioxochol-8-en-24-oic-acid [26]
**8**	25.83	529.2786	MS^1^：529.2786 [M − H]^−^, 511.2697 [M − H − 18(H_2_O)]^−^ MS^2^：511.2697→467.3350 [M − H − 18(H_2_O) − 44(CO_2_)]^−^, 437.3528[M − H − 18(H_2_O) − 44(CO_2_) − 30(2CH_3_)]^−^, 317.0999 [M − H − 18(H_2_O) − 194(pyrolysis fragments of D ring)]^−^ MS^3^：467.3350→423.3057 [M − H − 18(H_2_O) − 44(CO_2_) − 44(CO_2_)]^−^	Ganoderic acid C_6_ [26]
**9**	28.17	531.2941	MS^1^：531.2941 [M − H]^−^, 513.2853 [M − H − 18(H_2_O)]^−^ MS^2^：513.2853→469.3372 [M − H − 18(H_2_O) − 44(CO_2_)]^−^, 454.2572 [M − H − 18(H_2_O) − 44(CO_2_) − 15(CH_3_)]^−^, 436.2994 [M − H − 18(H_2_O) − 44(CO_2_) − 18(H_2_O) − 15(CH_3_)]^−^, 301.1445 [M − H − 18(H_2_O) − 18(H_2_O) − 194(pyrolysis fragments of D ring)]^−^ MS^3^：469.3372→451.3330 [M − H − 18(H_2_O) − 44(CO_2_) − 18(H_2_O)]^−^, 265.0820 [M − H − 18(H_2_O) − 44(CO_2_) − 204 (pyrolysis fragments of C ring)]^−^	Ganoderic acid G [26,31]
**10**	31.25	516.2992	MS^1^：516.2992 [M − H]^−^, 497.2901 [M − H − 18(H_2_O)]^−^ MS^2^：497.2901→453.2937 [M − H − 18(H_2_O) − 44(CO_2_)]^−^, 303.2104 [M − H − 18(H_2_O) − 194(pyrolysis fragments of D ring)]^−^, 287.2104 [M − H − 194(pyrolysis fragments of D ring) − 16(CH_4_)]^−^ MS^3^：453.2937→435.2029 [M − H − 44(CO_2_) − 36(2H_2_O)]^−^, 409.3284 [M − H − 18(H_2_O) − 44(CO_2_) − 44(CO_2_)]^−^, 249.0864 [M − H − 18(H_2_O) − 44(CO_2_) − 204(pyrolysis fragments of C ring)]^−^	Ganoderic acid B [26,30,31]
**11**	33.14	511.2698	MS^1^：511.2698 [M − H]^−^ MS^2^：511.2698→493.3167 [M − H − 18(H_2_O)]^−^, 467.3325 [M − H − 44(CO_2_)]^−^, 449.3569 [M − H − 18(H_2_O) − 44(CO_2_)]^−^, 434.2375 [M − H − 18(H_2_O) − 59(Ac-)]^−^ MS^3^：493.3167→245.1126 [M − H − 18(H_2_O) − 44(CO_2_) − 204 (pyrolysis fragments of C ring)]^−^ 147.0566 [M − H − 18(H_2_O) − 44(CO_2_) − 204 (pyrolysis fragments of C ring) − 98(pyrolysis fragments of A ring)]^−^	unknown
**12**	34.63	513.2588	MS^1^：513.2588 [M − H]^−^ MS^2^：513.2588→495.2083 [M − H − 18(H_2_O)]^−^, 451.2515 [M − H − 18(H_2_O) − 44(CO_2_)]^−^, 436.2632 [M − H − 18(H_2_O) − 59(Ac-)]^−^ MS^3^：495.2083→249.0978 [M − H − 18(H_2_O) − 36(2H_2_O) − 16(CH_4_) − 194(pyrolysis fragments of D ring)]^−^	Ganoderic acid AM_1_ [26,32]
**13**	38.02	573.3042	MS^1^：573.3042 [M − H]^−^, 555.2953 [M − H − 18(H_2_O)]^−^ MS^2^：555.2953→511.2890 [M − H − 18(H_2_O) − 44(CO_2_)]^−^, 496.3256 [M − H − 18(H_2_O) − 59(CH_3_COO-)]^−^ MS^3^：511.2890→265.0914 [M − H − 18(H_2_O) − 44(CO_2_) − 42(CH_2_=CO) − 204(pyrolysis fragments of C ring)]^−^ 496.3256→302.1797 [M − H − 18(H_2_O) − 59((CH_3_COO) − 194(pyrolysis fragments of D ring)]^−^	Ganoderic acid K [26]
**14**	40.45	457.2594	MS^1^：457.2594 [M − H]^−^ MS^2^：457.2594→442.4391 [M − H − 15(CH_3_)]^−^, 439.0501[M − H − 18(H_2_O)]^−^, 421.4436[M − H − 36(2H_2_O)]^−^ 395.3611 [M − H − 18(H_2_O) − 44(CO_2_)]^−^, 301.3354 [M − H − 138(pyrolysis fragments of D ring) − 18(H_2_O)]^−^	Lucidenic acid A [26]
**15**	44.49	515.3004	MS^1^：515.3004 [M − H]^−^ MS^2^：515.3004→497.2571 [M − H − 18(H_2_O)]^−^, 479.3175 [M − H − 36(2H_2_O)]^−^ MS^3^：497.2571→435.3353 [M − H − 18(H_2_O) − 18(H_2_O) − 44(CO_2_)]^−^, 303.1984 [M − H − 18(H_2_O) − 194(pyrolysis fragments of D ring)]^−^	Ganoderic acid A [26,30,31]
**16**	46.25	571.2893	MS^1^：571.2893 [M − H]^−^, 553.2797 [M − H − 18(H_2_O)]^−^ MS^2^：553.2797→511.2424 [M − H − 18(H_2_O) − 42(CH_2_=CO)]^−^, 481.3605 [M − H − 18(H_2_O) − 42(CH_2_=CO) − 30(2CH_3_)]^−^, MS^3^：511.2424→467.3026 [M − H − 18(H_2_O) − 42(CH_2_=CO) − 44(CO_2_)]^−^, 437.3870 [M – H − 18(H_2_O) − 42(CH_2_=CO) − 44(CO_2_) -30(2CH_3_)]^−^, 303.1073[M − H − 18(H_2_O) − 42(CH_2_=CO) − 194(pyrolysis fragments of D ring) − 14(CH_2_)]^−^, 301.1706[M – H − 18(H_2_O) − 42(CH_2_=CO) − 194(pyrolysis fragments of D ring) − 16(CH_4_)]^−^	Ganoderic acid H [26,33]
**17**	52.47	527.2637	MS^1^：527.2637 [M − H]^−^, 509.2544 [M − H − 18(H_2_O)]^−^ MS^2^：509.2544→465.2312 [M − H − 18(H_2_O) − 44(CO_2_)]^−^, 435.2996 [M − H − 18(H_2_O) − 44(CO_2_) − 30(2CH_3_)]^−^, 301.2139 [M − H − 18(H_2_O) − 194(pyrolysis fragments of D ring) − 14(CH_2_)]^−^, 299.1358 [M − H − 18(H_2_O) − 194(pyrolysis fragments of D ring) − 16(CH_4_)]^−^	12-hydroxy-3,7,11,15,23-pentaoxo-lanost-8-en-26-oic acid [26]
**18**	62.71	615.2795	MS^1^：615.2795 [M − H]^−^, 597.3021 [M − H − 18(H_2_O)]^−^ MS^2^：597.3021→553.2849 [M − H − 18(H_2_O) − 44(CO_2_)]^−^, 511.2561 [M − H − 18(H_2_O) − 44(CO_2_) − 42(CH_2_=CO)]^−^, 493.2861 [M − H − 18(H_2_O) − 88(2CO_2_) − 16(CH_4_)]^−^, 467.4220[M − H − 18(H_2_O) − 88(2CO_2_) − 42(CH_2_=CO)]^−^ MS^3^：553.2849→509.1722 [M − H − 18(H_2_O) − 44(CO_2_) − 44(CO_2_)]^−^, 479.1404 [M − H − 18(H_2_O) − 44(CO_2_) − 44(CO_2_) − 30(2CH_3_)]^−^,449.4641 [M − H − 18(H_2_O) − 44(CO_2_) − 44(CO_2_) − 42(CH_2_=CO) − 18(H_2_O)]^−^	12,15-bis(acetyloxy)-3-hydroxy-7,11,23-trioxo-lanost-8-en-26-oic-acid [26]
**19**	69.36	513.2836	MS^1^：513.2836 [M − H]^−^, 495.2746 [M − H − 18(H_2_O)]^−^ MS^2^：495.2746→451.3033 [M − H − 18(H_2_O) − 44(CO_2_)]^−^, 436.2344 [M − H − 18(H_2_O) − 44(CO_2_) − 15(CH_3_)]^−^, 301.1673 [M − H − 18(H_2_O) − 194(pyrolysis fragments of D ring)]^−^, 285.1029 [M − H − 18(H_2_O) − 194(pyrolysis fragments of D ring) − 14(CH_2_)]^−^, MS^3^：451.3033→433.3118 [M − H − 18(H_2_O) − 44(CO_2_) − 18(H_2_O)]^−^, 407.2886[M − H − 18(H_2_O) − 44(CO_2_) − 44(CO_2_)]^−^, 247.0793 [M − H − 18(H_2_O) − 44(CO_2_) − 204(pyrolysis fragments of C ring)]^−^	Ganoderic acid D [26,30]
**20**	75.66	511.2693	MS^1^：511.2693 [M − H]^−^ MS^2^：511.2693→493.2604 [M − H − 18(H_2_O)]^−^, 449.2799[M − H − 18(H_2_O) − 44(CO_2_)]^−^ MS^3^：493.2604→299.2487 [M − H − 18(H_2_O) − 194(pyrolysis fragments of D ring)]^−^ 449.2799→434.2175 [M − H − 18(H_2_O) − 44(CO_2_) − 15(CH_3_)]^−^, 419.3584 [M − H − 18(H_2_O) − 44(CO_2_) − 30(2CH_3_)]^−^	Ganoderic acid F [26]
**21**	77.24	499.3067	MS^1^：499.3067 [M − H]^−^ MS^2^：499.3067→481.3056 [M − H − 18(H_2_O)]^−^, 437.3787 [M − H − 18(H_2_O) − 44(CO_2_)]^−^, MS^3^：481.3056→287.2167 [M − H − 18(H_2_O) − 194(pyrolysis fragments of D ring)]^−^, 437.3787→419.2850 [M − H − 18(H_2_O) − 44(CO_2_) − 18(H_2_O)]^−^	Ganolucidic acid D [26]
**22**	80.47	569.2731	MS^1^：569.2731 [M − H]^−^, 551.0040 [M − H − 18(H_2_O)]^−^ MS^2^：551.0040→509.2411 [M − H − 18(H_2_O) − 42(CH_2_=CO)]^−^, 479.2818[M − H − 18(H_2_O) − 42(CH_2_=CO) − 30(2CH_3_)]^−^, 317.2806 [M − H − 204 (pyrolysis fragments of C ring) − 30(2CH_3_)]^−^ MS^3^：509.2411→465.2256 [M − H − 18(H_2_O) − 42(CH_2_=CO) − 44(CO_2_)]^−^, 435.3218 [M − H − 18(H_2_O) − 42(CH_2_=CO) − 44(CO_2_) − 30(2CH_3_)]^−^, 301.2180[M − H − 18(H_2_O) − 42(CH_2_=CO) − 194(pyrolysis fragments of D ring) − 14(CH_2_)]^−^	12-acetoxyganoderic acid F [26,27]
**23**	81.87	513.2857	MS^1^：513.2857 [M − H]^−^ MS^2^：513.2857→451.2750 [M − H − 18(H_2_O) − 44(CO_2_)]^−^, 436.3795 [M − H − 18(H_2_O) − 44(CO_2_) − 15(CH_3_) ]^−^, 305.2700 [M − H − 194(pyrolysis fragments of D ring) − 14(CH_2_)]^−^, 251.1266 [M − H − 44(CO_2_) − 204(pyrolysis fragments of C ring) − 14(CH_2_)]^−^ MS^3^：451.2750→421.2310 [M − H − 18(H_2_O) − 44(CO_2_) − 30(2CH_3_)]^−^, 403.253 [M − H − 18(H_2_O) − 44(CO_2_) − 30(2CH_3_) − 18(H_2_O)]^−^	Ganoderic acid J [26]
**24**	86.30	497.2899	MS^1^：497.2899 [M − H]^−^ MS^2^：497.2899→479.2302 [M − H − 18(H_2_O)]^−^, 453.2728 [M − H − 44(CO_2_)]^−^, 435.2746 [M − H − 18(H_2_O) − 44(CO_2_)]^−^, 285.1586 [M − H − 18(H_2_O − 194(pyrolysis fragments of D ring)]^−^	Ganoderic acid GS [32]
**25**	88.22	483.3108	MS^1^：483.3108 [M-H]^−^ MS^2^：483.3108→467.2955 [M − H − 16(CH_4_)]^−^, 465.3409 [M − H − 18(H_2_O)]^−^, 439.3409 [M − H − 44(CO_2_)]^−^, 421.3387 [M − H − 18(H_2_O) − 44(CO_2_)]^−^, 385.1546 [M − H − 98(pyrolysis fragments of A ring)]^−^, 345.2003 [M − H − 138(pyrolysis fragments of B ring)]^−^, 315.1342 [M − H-178(pyrolysis fragments of D ring)], 287.1245 [M − H − 138(pyrolysis fragments of B ring) − 18(H_2_O)]^−^ MS^3^：345.2003→301.2150 [M − H − 138(pyrolysis fragments of B ring) − 44(CO_2_)]^−^, 271.0611 [M − H − 138(pyrolysis fragments of B ring) − 44(CO_2_) − 30(2CH_3_)]^−^, 269.1784 [M − H − 138(pyrolysis fragments of B ring) − 44(CO_2_) − 32(2CH_4_)]^−^	3,7-oxo-12-hydroxy-ganoderic acid DM [27,32]
**26**	91.31	529.3177	MS^1^：529.3177 [M − H]^−^ MS^2^：529.3177→511.3445 [M − H − 18(H_2_O)]^−^, 493.3448 [M − H − 36(2H_2_O)]^−^, 467.3685 [M − H − 18(H_2_O) − 44(CO_2_)]^−^, 299.1341 [M − H − 36(2H_2_O) − 194(pyrolysis fragments of D ring)]^−^ MS^3^：467.3685→449.3226 [M − H − 18(H_2_O) − 44(CO_2_) − 18(H_2_O)]^−^, 419.1971 [M − H − 18(H_2_O) − 44(CO_2_) − 18(H_2_O) − 30(2CH_3_)]^−^, 263.3528 [M − H − 18(H_2_O) − 44(CO_2_) − 204(pyrolysis fragments of C ring)]^−^, 247.0979 [M − H − 18(H_2_O) − 44(CO_2_) − 204(pyrolysis fragments of C ring) − 16(CH_4_)]^−^	12-hydroxyganoderic acid D [26]
**27**	91.83	613.3005	MS^1^：613.3005 [M − H]^−^, 595.2902 [M − H − 18(H_2_O)]^−^ MS^2^：595.2902→553.2996 [M − H − 18(H_2_O) − 42(CH_2_=CO)]^−^, 523.2399 [M − H − 18(H_2_O) − 44(CO_2_) − 28(2CH_2_))]^−^, 509.3708 [M − H − 18(H_2_O) − 44(CO_2_) − 42(CH_2_=CO)]^−^ MS^3^：553.2996→479.2277 [M − H − 18(H_2_O) − 42(CH_2_=CO) − 44(CO_2_) − 30(2CH_3_)]^−^, 465.3148[M − H − 18(H_2_O) − 42(CH_2_=CO) − 88(2CO_2_)]^−^, 345.2563 [M − H − 18(H_2_O) − 42(CH_2_=CO) − 194(pyrolysis fragments of D ring) − 14(CH_2_)]^−^, 343.3474 [M − H − 18(H_2_O) − 42(CH_2_=CO) − 194(pyrolysis fragments of D ring) − 16(CH_4_)]^−^	3-acetylganoderic acid H [26]
**28**	91.30	570.0023	MS^1^：570.0023 [M − H]	Unknown
**29**	93.34	483.3266	MS^1^：483.3266 [M − H]^−^ MS^2^：483.3266→465.3160 [M − H − 18(H_2_O)]^−^, 447.2954 [M − H − 36(2H_2_O)]^−^, 439.4073453.2728 [M − H − 44(CO_2_)]^−^, 421.4003 [M − H − 18(H_2_O) − 44(CO_2_)]^−^, 361.1981 [M − H − 18(H_2_O) − 44(CO_2_) − 60(CH_3_COOH)]^−^, 255.1103 [M − H − 178(pyrolysis fragments of D ring) − 18(H_2_O) − 32(2CH_4_)]^−^	15-hydroxyganoderic acid DM [32]
**30**	95.05	525.3211	MS^1^：525.3211 [M − H]^−^ MS^2^：525.3211→483.2451 [M − H − 42(CH_2_=CO)]^−^, 439.4126 [M − H − 42(CH_2_=CO) − 44(CO_2_)]^−^, 421.4462 [M − H − 42(CH_2_=CO) − 44(CO_2_) − 18(H_2_O)]^−^,329.4416 [M − H − 18(H_2_O) − 178(pyrolysis fragments of D ring)]^−^ MS^3^：483.2451→465.3002 [M − H − 42(CH_2_=CO) − 18(H_2_O)]^−^, 287.2225 [M − H − 42(CH_2_=CO) − 18(H_2_O) − 178(pyrolysis fragments of D ring)]^−^, 269.1860 [M − H − 42(CH_2_=CO) − 36(2 H_2_O) − 178(pyrolysis fragments of D ring)]-	3,7-oxo-12-acetylganoderic acid DM [26]
**31**	96.23	571.2204	MS^1^：571.2204 [M − H]^−^	Unknown
**32**	97.07	499.3419	MS^1^：499.3419 [M − H]^−^ MS^2^：499.3419→481.2946 [M − H − 18(H_2_O)]^−^, 455.0124 [M − H − 44(CO_2_)]^−^ , 437.2764 [M − H − 18(H_2_O) − 44(CO_2_)]^−^ 287.0924 [M − H − 194(pyrolysis fragments of D ring) − 18(H_2_O)]^−^	Ganolucidic acid A [26]
**33**	99.83	467.3156	MS^1^：467.3156 [M − H]^−^ MS^2^：467.3156→449.3837 [M − H − 18(H_2_O)]^−^, 423.3398 [M − H − 44(CO_2_)]^−^, 383.0190 [M − H − 84(2CH_2_=CO)]^−^, 257.1906 [M − H − 178 (pyrolysis fragments of D ring) − 32(2CH_4_)]^−^ MS^3^：423.3398→407.2750 [M − H − 44(CO_2_) − 16(CH_4_)]^−^, 337.3115 [M − H − 44(CO_2_) − 44(CO_2_) − 42(CH_2_=CO)]^−^, 311.2945 [M − H − 44(CO_2_) − 98(pyrolysis fragments of A ring) − 14(CH_2_)]^−^	Ganoderic acid DM [32]
**34**	103.86	401.0025	MS^1^：401.0025 [M - H]^−^ MS^2^：401.0025→383.1729 [M − H − 18(H_2_O)]^−^, 344.2189 [M − H − 42(CH_2_=CO) − 15(CH_3_)]^−^, 303.2025 [M − H − 18(H_2_O) − 80(pyrolysis fragments of D ring)]^−^	Lucidone A [32]
**35**	111.95	453.3369	MS^1^：453.3369 [M − H]^−^ MS^2^：453.3369→435.2218 [M − H − 18(H_2_O)]^−^, 409.4311 [M − H − 44(CO_2_)]^−^, 393.2309 [M − H − 60 (CH_3_COOH)]^−^, 391.4413 [M − H − 18(H_2_O) − 44(CO_2_)]^−^, 207.1283[M − H − 42(CH_2_=CO) − 204(pyrolysis fragments of C ring)]^−^ MS^3^：393.2309→375.2531 [M − H − 60 (CH_3_COOH) − 18(H_2_O)]^−^, 359.2667 [M − H − 60 (CH_3_COOH) − 18(H_2_O) − 16(CH_4_)]^−^	Ganoderic acid TR or Ganoderic acid Y [32]
**36**	116.41	495.2749	MS^1^：495.2749 [M − H]^−^ MS^2^：495.2749→477.4175 [M − H − 18(H_2_O)]^−^, 451.2777 [M − H − 44(CO_2_)]^−^, 436.2990 [M − H − 44(CO_2_) − 15(CH_3_)]^−^, 301.1088 [M − H − 194(pyrolysis fragments of D ring)]^−^, 285.1394 [M − H − 194(pyrolysis fragments of D ring) - 16(CH_4_)]^−^, 247.1259 [M − H − 44(CO_2_) − 204(pyrolysis fragments of C ring)]^−^	3,11,15-trioxochol-8-en-24-oic acid [26,27]
**37**	119.35	459.2901	MS^1^：459.2901 [M − H]^−^ MS^2^：459.2901→441.4392 [M − H − 18(H_2_O)]^−^, 423.2791 [M − H − 36(2H_2_O)]^−^, 397.6952 [M − H − 18(H_2_O) − 44(CO_2_)]^−^, 285.2697 [M − H − 36(2H_2_O) − 138(pyrolysis fragments of D ring)]^−^, 269.1612 [M − H − 36(2H_2_O) − 138(pyrolysis fragments of D ring) − 16(CH_4_)]^−^	7,15-dihydroxy-4,4,14-trimethyl-3,11-dioxochol-8-en-24-oic acid [26]
**a1**	13.31	527.2641	MS^1^：527.2641 [M − H]^−^ MS^2^：527.2641→509.2797 [M − H − 18(H_2_O)]^−^, 483.2253 [M − H − 44(CO_2_)]^−^, 465.2714 [M − H − 18(H_2_O) − 44(CO_2_)]^−^, 317.1736 [M − H − 18(H_2_O) − 192(pyrolysis fragments of D ring)]^−^MS3：465.2714→447.2611 [M − H − 18(H_2_O) − 44(CO_2_) − 18(H_2_O)] ^−^, 421.2402 [M − H − 18(H_2_O) − 44(CO_2_) − 44(CO_2_)]^−^	3,12-dihydroxy-4,4,14-trimethyl-7,11,15- trioxo-lanost-8,9,20,22-en-26-oic acid [26,27]
**a2**	13.71	511.3550	MS^1^：511.3550 [M − H]^−^ MS^2^：511.3550→469.3110 [M − H − 42(CH_2_=CO)]^−^, 467.2477[M − H − 44(CO_2_)]^−^, 425.3692[M − H − 42(CH_2_=CO) − 44(CO_2_)]^−^, 303.1880 [M − H − 192(pyrolysis fragments of D ring) − 16(CH_4_)]^−^	Ganoderic acid Mf [26,33]
**a3**	29.16	459.2763	MS^1^：459.2763 [M − H]^−^ MS^2^：459.2763→441.2818 [M − H − 18(H_2_O)]^−^, 423.3502 [M − H − 36(2H_2_O)]^−^, 397.4172 [M − H − 18(H_2_O) − 44(CO_2_)]^−^ 303.2930 [M − H − 18(H_2_O) − 138(pyrolysis fragments of D ring)]^−^, 289.2338 [M − H − 18(H_2_O) − 138(pyrolysis fragments of D ring) − 14(CH_2_)]^−^, 288.4626 [M − H − 18(H_2_O) − 138(pyrolysis fragments of D ring) − 15(CH_3_)]^−^	Lucidenic acid N [26]
**a4**	49.03	511.2703	MS^1^：511.2703 [M − H]^−^, 493.2587 [M − H − 18(H_2_O)]^−^ MS^2^：493.2587→478.3034 [M − H − 18(H_2_O) − 15(CH_3_)]^−^, 449.3233[M − H − 18(H_2_O) − 44(CO_2_)]^−^, 431.3262 [M − H − 18(H_2_O) − 44(CO_2_) − 18(H_2_O)]^−^, 301.0695 [M − H − 192(pyrolysis fragments of D ring)]^−^, 261.1931 [M − H − 204 (pyrolysis fragments of C ring) − 28 (CO)]^−^, 247.0212[M - H − 204 (pyrolysis fragments of C ring) − 42(CH_2_=CO)]^−^	Ganoderenic acid D [26]
**a5**	52.47	515.3007	MS^1^：515.3007 [M − H]^−^ MS^2^：515.3007→497.3394 [M − H − 18(H_2_O)]^−^, 453.2672 [M − H − 18(H_2_O) − 44(CO_2_)]^−^, 435.3178[M − H − 36(2H_2_O) − 44(CO_2_)]^−^ MS^3^：497.3394→435.3178 [M − H − 18(H_2_O) − 18(H_2_O) − 44(CO_2_)]^−^, 303.2353 [M − H − 18(H_2_O) − 194 (pyrolysis fragments of D ring)]^−^	Ganoderic acid δ [31,33]
**a6**	54.24	527.2637	MS^1^：527.2637 [M − H]^−^, 509.2544 [M - H - 18(H_2_O)]^−^ MS^2^：509.2544→479.1830 [M − H − 18(H_2_O) − 30(2CH_3_)]^−^, 465.2850 [M − H − 18(H_2_O) − 44(CO_2_)]^−^, 435.2603 [M − H − 18(H_2_O) − 44(CO_2_) − 30(2CH_3_)]^−^, 317.2471 [M − H − 18(H_2_O) − 192 (pyrolysis fragments of D ring)]^−^, 301.1240 [M − H − 18(H_2_O) − 192 (pyrolysis fragments of D ring) − 16(CH_4_)]^−^, 299.1788 [M − H − 18(H_2_O) − 192 (pyrolysis fragments of D ring) − 18(H_2_O)]^−^	Elfvingic acid A [26]
**a7**	69.32	513.2836	MS^1^：513.2836 [M − H]^−^, 495.2746 [M − H − 18(H_2_O) ^−^ MS^2^：495.2746→451.3008 [M − H − 18(H_2_O) − 44(CO_2_)]^−^, 437.3971 [M − H − 18(H_2_O) − 44(CO_2_) − 14(CH_2_)]^−^, 303.1641 [M − H − 18(H_2_O) − 192 (pyrolysis fragments of D ring)]^−^, 287.1062 [M − H − 18(H_2_O) − 192 (pyrolysis fragments of D ring) − 16(CH_4_)]^−^ MS^3^：451.3008→433.2937 [M − H − 18(H_2_O) − 44(CO_2_) − 18(H_2_O)]^−^, 407.3061 [M − H − 18(H_2_O)) − 44(CO_2_) − 44(CO_2_)]^−^, 247.0545 [M − H − 18(H_2_O) − 44(CO_2_) − 18(H_2_O) − 204 (pyrolysis fragments of C ring)]^−^	Ganoderenic acid B [26]
**a8**	79.87	513.2494	MS^1^：513.2836 [M - H]^−^ MS^2^：513.2494→471.1854 [M − H − 42(CH_2_=CO)]^−^, 456.3038 [M − H − 42(CH_2_=CO) − 15(CH_3_)]^−^, 453.1012 [M − H − 42(CH_2_=CO) − 18(H_2_O)]^−^, 435.2854 [M − H − 42(CH_2_=CO) − 36(2H_2_O)]^−^, 301.2219 [M − H − 42(CH_2_=CO) − 138 (pyrolysis fragments of D ring) − 32(2CH_4_)]^−^	Lucidenic acid D [26]
**a9**	88.41	555.2974	MS^1^：555.2974 [M - H]^−^ MS^2^：555.2974→537.0157 [M − H − 18(H_2_O)]^−^, 513.3628 [M − H − 42(CH_2_=CO)]^−^, 495.2735 [M − H − 18(H_2_O) − 42(CH_2_=CO)]^−^, 451.3274 [M − H − 18(H_2_O) − 42(CH_2_=CO) − 44(CO_2_)]^−^ MS^3^：513.3628→263.1146 [M - H − 42(CH_2_=CO) − 56(2CO) − 194 (pyrolysis fragments of D ring)]^−^, 249.3468 [M − H − 42(CH_2_=CO) − 18(H_2_O) − 42(CH_2_=CO) − 204 (pyrolysis fragments of C ring)]^−^, 247.0499 [M − H − 42(CH_2_=CO) − 18(H_2_O) − 44(CO_2_) − 204 (pyrolysis fragments of C ring)]^−^	Lucidenic acid GS-3 [32,33]
**a10**	124.88	471.3473	MS^1^：471.3473 [M − H]^−^ MS^2^：471.3473→435.4189 [M − H − 36 (2H_2_O)]^−^, 395.3422 [M − H − 32(2CH_4_) − 44(CO_2_)]^−^, 367.1648 [M − H − 44(CO_2_) - 60(CH_3_COOH)]^−^, 353.1996 [M − H − 44(CO_2_) − 60(CH_3_COOH) − 14(CH_2_)]^−^	unknown

**Table 3:** The chemical structures of the identified compounds.

No.	Chemical Name	Ty.	R1	R2	R3	R4	C=C	M
**1**	12-Hydroxyganoderic acid C_2_	A	β-OH	β-OH	α-OH	OH	-	534.3109
**3**	3-Acetylganoderenic acid K	A	β-OAc	β-OH	=O	β-OAc	^Δ^20, 22	613.2977
**4**	3,7,15-Trihydroxy-11,23-dioxolanost-8,16-dien-26-oic acid	A	β-OH	β-OH	β-OH	-	^Δ^16, 17	516.3011
**5**	Ganoderic acid C_2_	A	β-OH	β-OH	α-OH	H	-	518.3159
**6**	Ganolucidic acid B	A	β-OH	H	α-OH	H	-	502.3214
**7**	3-Hydroxy-4,4,14-trimethyl-7,11,15-trioxochol-8-en-24-oic-acid	B	β-OH	=O	=O	H	-	458.2592
**8**	Ganoderic acid C_6_	A	β-OH	=O	=O	β-OH	-	530.2786
**9**	Ganoderic acid G	A	β-OH	β-OH	=O	β-OH	-	532.2941
**10**	Ganoderic acid B	A	β-OH	β-OH	=O	H	-	516.2992
**12**	Ganoderic acid AM_1_	A	β-OH	=O	=O	H	-	514.2588
**13**	Ganoderic acid K	A	β-OH	β-OH	=O	β-OAc	-	574.3042
**14**	Lucidenic acid A	B	=O	β-OH	=O	H		458.2594
**15**	Ganoderic acid A	A	=O	β-OH	α-OH	H	-	516.3004
**16**	Ganoderic acid H	A	β-OH	=O	=O	β-OAc	-	572.2893
**17**	12-Hydroxy-3,7,11,15,23-pentaoxolanost-8-en-26-oic acid	A	=O	=O	=O	-OH	-	528.2637
**18**	12,15-Bis(acetyloxy)-3-hydroxy-7,11,23-trioxo-lanost-8-en-26-oic-acid	A	OH	=O	OAc	OAc	-	616.2795
**19**	Ganoderic acid D	A	=O	β-OH	=O	H	-	514.2836
**20**	Ganoderic acid F	A	=O	=O	=O	H	-	512.2693
**21**	Ganolucidic acid D	C	-	-	-	-	-	500.3067
**22**	12-Acetoxyganoderic acid F	A	=O	=O	=O	β-OAc	-	570.2731
**23**	Ganoderic acid J	A	=O	=O	α-OH	H	-	514.2857
**24**	Ganoderic acid GS	A	=O	=OH	=O	=O	-	498.2899
**25**	3,7-Oxo-12-hydroxy-ganoderic acid DM	D	=O	=O	H	OH	-	484.3108
**26**	12-Hydroxyganoderic acid D	A	=O	β-OH	=O	OH	-	530.3177
**27**	3-Acetylganoderic acid H	A	β-OAc	=O	=O	β-OAc	-	614.3005
**29**	15-Hydroxyganoderic acid DM	D	=O	H	-OH	H	-	484.3266
**30**	3,7-Oxo-12-acetylganoderic acid DM	D	=O	=O	-	β-OAc	-	526.3211
**32**	Ganolucidic acid A	A	=O	H	α-OH	H	-	500.3419
**33**	Ganoderic acid DM	D	=O	H	H	H	-	468.3156
**34**	Lucidone A	E	-	-	-	-	-	402.0025
**35**	Ganoderic acid TR	F	-	-	-	-	-	454.3369
	Ganoderic acid Y	G	β-OH	-	-	-	-	
**36**	3,11,15-Trioxochol-8-en-24-oic acid	A	=O	H	=O	H	-	496.2749
**37**	7,15-Dihydroxy-4,4,14-trimethyl-3,11-dioxochol-8-en-24-oic acid	B	=O	OH	OH	H	-	460.2901
**a1**	3,12-Dihydroxy-4,4,14-trimethyl-7,11,15-trioxolanost-8,9,20,22-en-26-oic acid	A	β-OH	=O	=O	β-OH	^Δ^20, 22	528.2641
**a2**	Ganoderic acid Mf	H	β-OAc	-	-	-	-	512.3550
**a3**	Lucidenic acid N	B	β-OH	β-OH	=O	H	-	460.2763
**a4**	Ganoderenic acid D	A	=O	β-OH	=O	H	^△^20, 22	512.2703
**a5**	Ganoderic acid δ	C	-	-OH	-	H	^-^	516.3007
**a6**	Elfvingic acid A	A	=O	=O	β-OH	α-OH	^Δ^20, 22	528.2637
**a7**	Ganoderenic acid B	A	β-OH	β-OH	=O	H	^Δ^20, 22	514.2836
**a8**	Lucidenic acid D	B	=O	=O	=O	β-OAc	-	514.2494
**a9**	Lucidenic acid GS-3	A	β-OH	β-OH	=O	β-OAc	-	556.2974

**Table 4:** Factor loading matrix of the testing samples.

Peak No.	Principal Component Values					
	PC1	PC2	PC3	PC4	PC5	PC6
**1**	0.058	0.077	−0.014	−0.087	−0.025	0.007
**2**	−0.018	−0.012	−0.087	0.074	0.407	0.008
**3**	0.092	0.006	−0.059	0.024	−0.079	0.058
**4**	0.078	0.040	−0.018	−0.077	0.037	0.053
**5**	0.019	−0.050	−0.043	0.307	−0.017	0.117
**6**	−0.010	0.096	0.062	0.048	−0.095	0.242
**7**	0.041	0.079	−0.157	0.057	0.035	0.121
**8**	0.057	−0.033	0.051	0.046	−0.051	0.147
**9**	0.079	−0.067	−0.024	0.077	0.085	0.048
**10**	0.096	−0.025	−0.044	0.025	−0.023	0.019
**11**	0.077	−0.050	0.078	−0.080	0.040	0.046
**12**	0.015	0.090	0.070	−0.074	0.006	0.162
**13**	0.057	0.011	0.032	0.004	0.019	0.386
**14**	0.078	−0.047	0.037	0.023	−0.008	0.072
**15**	−0.003	0.060	0.064	−0.033	0.076	0.075
**16**	0.042	−0.054	0.034	−0.089	0.164	0.259
**17**	0.049	−0.062	0.115	−0.069	0.117	0.068
**18**	−0.054	−0.005	−0.049	0.290	0.054	0.013
**19**	0.043	−0.006	−0.025	0.167	0.064	0.177
**20**	−0.017	−0.026	0.115	−0.069	0.117	0.068
**21**	−0.021	0.039	0.019	0.101	0.077	0.049
**22**	−0.015	0.050	0.015	0.099	−0.056	0.093
**23**	0.000	0.128	−0.153	0.023	0.095	0.043
**24**	0.032	0.002	0.016	−0.086	0.139	0.182
**25**	−0.018	0.106	0.025	−0.008	−0.100	0.012
**26**	−0.031	0.058	−0.061	−0.011	0.206	0.130
**27**	0.078	0.069	−0.070	−0.029	−0.048	0.054
**28**	−0.035	0.055	0.123	−0.071	−0.051	0.021
**29**	−0.029	0.065	0.031	0.103	−0.135	0.052
**30**	0.085	0.048	−0.050	−0.082	−0.027	0.007
**31**	0.075	0.012	−0.020	0.025	−0.052	0.062
**32**	−0.049	0.042	0.239	−0.059	−0.126	0.241
**33**	−0.040	0.069	−0.029	−0.098	0.186	0.118
**34**	−0.007	0.176	0.028	−0.131	−0.076	0.343
**35**	0.029	−0.041	0.059	0.159	−0.182	0.239
**36**	−0.020	−0.039	0.203	0.040	−0.111	0.004
**37**	0.068	−0.003	−0.016	0.056	−0.109	0.220

**Table 5:** The regions of origin of the 15 samples.

No.	Region	No.	Region
**S1**	Haikou, Hainan	**S9**	Huangshan, Anhui
**S2**	Baotou, Neimemg	**S10**	Jinzhai, Anhui
**S3**	Taishan, Shandong	**S11**	Xinyang, Henan
**S4**	Jiaxing, Shandong	**S12**	Dali, Yunnan
**S5**	Jilin, Jilin	**S13**	Tianlin, Guangxi
**S6**	Changbaishan, Jilin	**S14**	Shanghai
**S7**	Changchun, Jilin	**S15**	Fuzhou, Fujian
**S8**	Jingzhou, Hunan		

## Supplementary Materials

The supplementary materials are available online.
